# Impact of Diabetes Mellitus and Its Comorbidities on Elderly Patients Hospitalized in Internal Medicine Wards: Data from the RePoSi Registry

**DOI:** 10.3390/healthcare10010086

**Published:** 2022-01-03

**Authors:** Christiano Argano, Giuseppe Natoli, Salvatore Mularo, Alessandro Nobili, Marika Lo Monaco, Pier Mannuccio Mannucci, Francesco Perticone, Antonello Pietrangelo, Salvatore Corrao

**Affiliations:** 1 Internal Medicine Department iGR, National Relevance Hospital Trust, ARNAS Civico, Di Cristina e Benfratelli, 90127 Palermo, Italy; peppenatoli@gmail.com (G.N.); s.mularo@gmail.com (S.M.); marika.lomonaco@hotmail.it (M.L.M.); salvatore.corrao@unipa.it (S.C.); 2 Department of Neuroscience, IRCCS Istituto di Ricerche Farmacologiche Mario Negri, 20156 Milan, Italy; alessandro.nobili@marionegri.it; 3 Scientific Direction, IRCCS Foundation Ca’ Granda Ospedale Maggiore Policlinico, 20122 Milan, Italy; piermannuccio.mannucci@policlinico.mi.it; 4 Department of Medical and Surgical Sciences, University Magna Graecia of Catanzaro, 88100 Catanzaro, Italy; perticone@unicz.it; 5 Department of Internal Medicine II, Centre for Hemochromatosis, University of Modena and Reggio Emilia Policlinico, 41100 Modena, Italy; antonello.pietrangelo@unimore.it; 6 Dipartimento di Promozione della Salute, Materno Infantile, Medicina Interna e Specialistica di Eccellenza “G. D’Alessandro” (PROMISE), University of Palermo, 90127 Palermo, Italy

**Keywords:** diabetes, comorbidities, heart rate, cancer, male sex, in-hospital mortality, 1-year mortality

## Abstract

Background: Currently, diabetes represents the seventh leading cause of death worldwide, with a significant economic burden. The number and severity of comorbidities increase with age, and are identified as important determinants that influence the prognosis. We aimed to investigate comorbidities and outcomes in a cohort of hospitalized elderly patients affected by diabetes. Methods: In this observational study, we retrospectively analyzed data collected from the REgistro dei pazienti per lo studio delle POlipatologie e politerapie in reparti della rete Simi (RePoSi) registry. Socio-demographic, clinical characteristics, and laboratory findings were considered. The association between variables and in-hospital and 1-year follow-up were analyzed. Results: Among 4708 in-patients, 1378 (29.3%) had a diagnosis of diabetes. Patients with diabetes had more previous hospitalization, a clinically significant disability, and more need for a urinary catheter in comparison with subjects without diabetes. Patients affected by diabetes took more drugs, both at admission, at in-hospital stay, at discharge, and at 1-year follow-up. Thirty-five comorbidities were more frequent in patients with diabetes, and the first five were hypertension (57.1%), ischemic heart disease (31.4%), chronic renal failure (28.8%), atrial fibrillation (25.6%), and chronic obstructive pulmonary disease (22.7%). Heart rate was an independent predictor of in-hospital mortality. At 1-year follow-up, cancer and male sex were strongly independently associated with mortality. Conclusions: Our findings showed the severity of the impact of diabetes and its comorbidities in the real life of internal medicine and geriatric wards, and provide data to be used for a better tailored management of elderly in-patients with diabetes.

## 1. Introduction

Diabetes represents a worldwide epidemic with high economic and social costs. Its burden is increasing, with an estimated prevalence among adults rising from 151 million in 2000 to 536.6 million in 2021 with a prevalence of 10.5% for adults aged 20 to 79 years [1]. Furthermore, the global burden of diabetes among elderly patients is expected to increase in the coming years due to the reduced physical activity related to type 2 diabetes mellitus, unhealthy diets, rising incidences of type 1 diabetes, and aging of the world population [2]. Currently, 136 million of people aged 65 years and older have diabetes worldwide [1]. It is estimated that the number of people aged 65 years or older with diabetes will reach 195.2 million by 2030, and 276.2 million by 2045 [2]. In the United States, 10.5% of people of all ages had diabetes, and 26.8% of people aged 65 years or older are diagnosed with diabetes [3]. In Europe, close to 9% of the regional population aged between 20 and 79 years has diabetes [4]. In Italy, the prevalence ranges from 5% to 26%, with the highest percentage equal to 66.3% among older subjects (over 65 years) [5]. According to the International Diabetes Federation, diabetes accounted for at least 760 billion in medical costs in 2019, about 10% of total global medical expenditures. [6] Comorbidities are important determinants of diabetes burden in terms of the considerable impact on the patient’s quality of life, health status, hospitalization and outcomes. In the elderly, diabetes is often accompanied by comorbidities. About 60% of elderly subjects with diabetes have one comorbidity, whereas 40% have four or more comorbid chronic conditions [7]. We previously published data from the RePoSi registry that evidenced clinical impact of comorbidities according to gender differences [8,9] and different diseases in the elderly population hospitalized in internal medicine and geriatric Italian wards [10,11]. Recently, we showed that one of the stronger predictors of in-hospital mortality for older patients admitted in general wards was a glycemia level ≥250 mg/dL [12]. Given this background, the aim of our study was to assess comorbidities and prognostic factors for in-hospital and post-discharge 1-year mortality in a cohort of elderly patients with diabetes hospitalized in internal medicine and geriatric wards participating in the RePoSi registry study.

## 2. Materials and Methods

### 2.1. Data Collection and Study Population

Retrospectively, we analyzed the collected data within the frame of the RePoSi project in the recruitment weeks of 2010, 2012, 2014, and 2016. RePoSi is an independent and collaborative registry, organized by the Italian Society of Internal Medicine (SIMI), IRCCS Ca’ Granda Maggiore Policlinico Hospital Foundation, and the Mario Negri Institute for Pharmacological Research, that was made up to recruit, monitor, and evaluate hospitalized older adults ≥65 years admitted to 102 Italian internal medicine and geriatric wards, with data coming from each single medical record, and collected every 2 years from 2008. The project’s design has been previously described in detail [10]. Briefly, patients were eligible for RePoSi if: (1) they were admitted to one of the participating regional internal medicine wards during the four index weeks chosen for recruitment (one in February, one in June, one in September, and one in December); (2) their age was 65 years or older; (3) they gave informed consent. Each ward had to enroll at least ten consecutive eligible patients during each index week, recording data on socio-demographic details, the main reason for admission and comorbidities, diagnoses, treatment (including all drugs taken at hospital admission, and those recommended at discharge), clinical events during hospitalization, and outcome. During those weeks, all participating centers had to complete the registration of all patients admitted, indicating those who were consecutively enrolled. For patients who were excluded, the reason had to be given. Also, data on mortality or any new hospitalization were collected, with a telephone interview performed by a physician to the patient or his/her relatives, 3 and 12 months after hospital discharge. Then, a final database was created and checked by the Mario Negri Institute for Pharmacological Research. All patients with and without diabetes were included in the present study analysis. All patients provided informed consent. Data were collected in full compliance with the Italian law on personal data protection, and the RePoSi study was approved by the Ethics Committee of each participating center.

### 2.2. Socio-Demographic and Clinical Characteristics

Socio-demographic variables, such as age class, marital status, living arrangement, and need for assistance in daily living, were considered, along with laboratory findings in patients with diabetes compared to the ones without it. The following clinical characteristics were evaluated: cognitive status and mood disorders (by the Short-Blessed-Test (SBT) [13] and the Geriatric-Depression-Scale (GDS) [14], respectively); performance in activities of daily living at hospital admission (measured by means of the Barthel Index (BI) [15]); severity and comorbidity index (assessed by the Cumulative-Illness-Rating-Scale (CIRS-s and CIRS-c, respectively)) [16]; glomerular filtration rate (GFR) (using the Chronic Kidney Disease Epidemiology Collaboration formula) [17]; length of hospital stay; drug prescriptions (at admission, at discharge, at 3 and 12 months follow-up); destination at discharge; and in-hospital and 1-year mortality rate. The association between variables and in-hospital and 1-year mortality was analyzed.

### 2.3. Statistical Analysis

Quantitative variables were summarized as mean (95% confidence intervals), and categorical variables as percentage. Patients with significant disability were described according to a BI score of ≤40, as previously published [18]. However, in all logistic analyses, the BI score was used to avoid the loss of information. Fisher’s exact test for contingency tables, a Z-test, and a non-parametric Mann–Whitney-U-test were used when appropriate. A multivariate logistic analysis was used to assess the relationship between variables and in-hospital and 1-year follow-up mortality. Heart rate, glomerular filtration rate, and BI score were input in a logistic analysis by ten points (which means that the odds ratio measured the variation by 10 heartbeats for heart rate, 10 mL/min for eGFR, and 10 points for BI). Variables were chosen according to the Hosmer–Lemeshow methodology, as previously published [19]. After univariate analysis, only variables with a *p* < 0.20 were included in the final model; then, through a backward process, variables were excluded until a significance level of *p* < 0.20 was reached for each variable. The Hosmer–Lemeshow test is a goodness of fit test of the regression model which calculates if the observed event rates match the expected event rates in population subgroups. The application of the Hosmer–Lemeshow test is a measure of how well the model fits the data without any choice of variables by the researcher to put into the multivariate. A two-tailed *p* < 0.05 was considered statistically significant. Stata Statistical Software 2016, Release14 (Stata-Corp, College-Station, TX, USA) was used for database management and all the analyses.

## 3. Results

**Figure 1:**
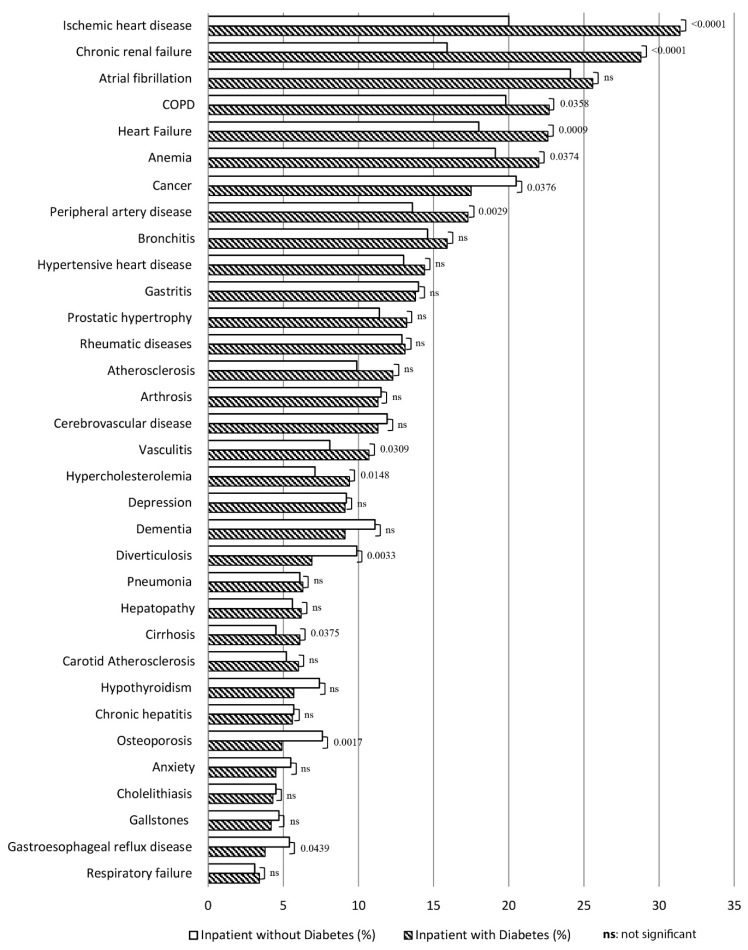
The most frequent clinical diagnoses (as percentage) in the RePoSi population according to diabetes categorization. Hypertension was the first diagnosis in patients with diabetes (57.1%) vs. patients without diabetes (54%) (*p* = 0.0783). It is not plotted because the value was too large, and would have altered the graphic representation.

**Figure 2:**
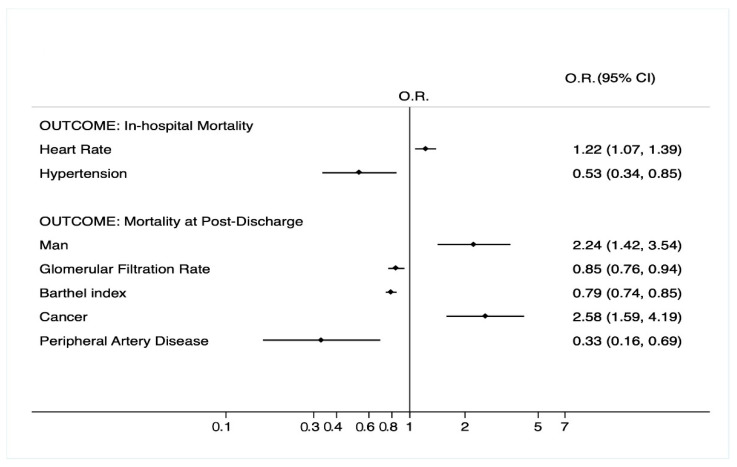
Multivariate analysis in patients with diabetes according to in-hospital and 1-year mortality. OR, odds ratio; 95% CI, 95% confidence interval. Glomerular filtration rate calculated by CKD-EPI formula; glomerular filtration rate is referred to values every 10 mL/min; heart rate is referred to values every 10 heartbeats; Barthel Index is referred to values every 10 points. Only the final model is shown according to Hosmer–Lemeshow methodology. For the selection of variables, see the Statistical Analysis section.

During the recruitment period, 4708 inpatients were eligible for this analysis; 1378 (29.3%) presented with a diagnosis of diabetes. Among them, 56.2% were men, with a mean age of 78.4 years. Table 1 shows the demographic characteristics and modifiable risk factors of the two study groups. Interestingly, 43% of in-patients with diabetes had a history of previous hospitalizations compared to only one third of non-diabetic inpatients (*p* = 0.0046). A significantly higher proportion of subjects with diabetes were more often obese (*p* < 0.0001 for all three class of obesity). Laboratory and clinical characteristics of inpatients with and without diabetes are shown in Table 2. Subjects with diabetes had a lower glomerular filtration rate (*p* < 0.0001), a lower BI Score (*p* = 0.0019), and 29.3% needed a urinary catheter (*p* = 0.0023) in comparison with those without diabetes. The group of patients with diabetes had a significantly higher CIRS for the evaluation of both severity and comorbidity indexes (*p* < 0.0001), and they took more drugs at hospital admission, during hospitalization, at hospital discharge, and at 1-year follow-up (*p* < 0.0001). Overall, disease distribution showed that arterial hypertension, ischemic heart disease, chronic renal failure, atrial fibrillation, COPD, heart failure, anemia, cancer, peripheral artery disease, and bronchitis were more frequent in patients with diabetes (Figure 1). As shown in Table 3, subjects with diabetes had a longer hospital stay, and higher in-hospital and 1-year mortality, albeit not significantly. When we assessed independent predictors of mortality, running a univariate analysis (see Appendix A) and then a multivariate analysis, heart rate (OR 1.22, 95% CI 1.07–1.39) was the strongest predictor of mortality at in-hospital mortality, whereas hypertension (OR 0.53, 95%CI 0.34–0.85) was protective. At 1-year follow-up, cancer (OR 2.58, 95% CI 1.59–4.19) and male sex (OR 2.24, 95% CI 1.42–3.54) were the strongest predictors of mortality, whereas peripheral artery disease (OR 0.33, 95% CI 0.16–0.69) was protective. Renal function and disability score were inversely related to mortality (OR 0.85, 95% CI 0.76–0.94; OR 0.79, 95% CI 0.74–0.85, respectively) (Figure 2).

## 4. Discussion

This study has assessed the distribution of comorbidities, and the occurrence of short and long outcomes in a hospitalized elderly population with diabetes admitted to the wards of the frame of the RePoSi registry, with the aim to evaluate whether diabetic subjects act differently from individuals without diabetes. Although comorbid chronic conditions are increasingly identified as important factors in diabetes management [20], there is a lack of research specifically designed to assess the relationship between comorbidities and the short-term and long-term outcomes of subjects with diabetes admitted to internal and geriatric wards. This study highlighted the role of heart rate, which significantly increased mortality during in-hospital admission. In fact, the rise of 10 heartbeats increased the risk of death by 22%. Our results agree with previous studies that have shown a relationship between higher resting heart rate and major cardiovascular events, cardiovascular death, and all-cause mortality in patients with type 2 diabetes mellitus, particularly in individuals with previous macrovascular complications [21]. Moreover, subjects affected by diabetes with a higher resting heart rate have a greater prevalence of microalbuminuria, and a new onset of nephropathy and retinopathy [22]. Regarding hypertension, it is well known that elevated blood pressure is considered an important additive risk factor in subjects affected by type 2 diabetes, increasing the risk of morbidity and mortality [23], and recent reviews of meta-analyses and large observational studies regarding the blood pressure targets to pursue in patients with diabetes showed evidence definitively against a reduction of systolic blood pressure lower than 120 mmHg [24]. According to Thomopoulos and Emdin, respectively [25,26], a sustained BP < 130/80 mmHg can be associated with an increased risk of cardiac events in high-risk patients with type 2 diabetes. Our findings are in line with previous studies that showed the protective role of systolic blood pressure regarding in-hospital and 3-month mortalities, and demonstrated an inverse association between higher blood pressure and mortality in the oldest patients [27,28]. Moreover, recent data showed that in contrast to the general population, in frail elderly patients, increased blood pressure is associated with reduced mortality. In the PARTAGE study [29], which assessed all-cause mortality according to systolic blood pressure levels achieved (target systolic blood pressure < 130 mm Hg), there was a higher risk of mortality in frail octogenarians who had lower systolic blood pressure. It is worth mentioning that in elderly patients with heart failure, there is no clear evidence to justify a single blood pressure target in patients with established heart failure. [30]. A possible explanation lies in the fact that high blood pressure is necessary to ensure sufficient organ perfusion in elderly people, who are likely to have significant vascular damage [31,32]. Another important finding concerns the crucial role of cancer and male sex at 1-year follow-up. Cancer is the strongest comorbidity associated with the risk of mortality at 1-year follow-up. This finding is consistent with previous studies which showed a strongly association between diabetes and several tumors, particularly pancreatic, liver, breast, kidney, bladder, endometrial, colorectal, and head and neck cancers [33]. In this sense, hyperglycemia induces a proliferative, anti-apoptotic, and metastatic effect [34], and hyperinsulinemia and obesity promote tumorigenesis [35,36]. Regarding the relationship between sex and mortality, our results are apparently in contrast with recent literature, which showed that women have greater increases of cardiovascular risk, myocardial infarction, and stroke mortality in comparison with men [37], and that diabetes represents a stronger risk factor for vascular disease in women than men [38]. Our study took into account a group of very elderly in-patients hospitalized in internal medicine wards with multiple chronic conditions and a higher percentage of male in-patients. In this regard, our analysis was in line with previous studies that highlighted a male profile more inclined to be affected by diabetes mellitus, coronary artery disease, COPD, and malignancy [5,6] in the group of patients admitted to the internal and geriatric wards. With regard to the role of BI, data from the RePoSi registry showed that BI ≤ 40, CIRS-SI and glycemia level ≥ 250 mg/dL were the stronger predictors of in-hospital mortality for older patients admitted in general wards [12]. Elderly in-patients with pneumonia with a clinically significant disability had a higher mortality risk [18]. Moreover, Gofir and colleagues showed that hyperglycemia was an independent factor of functional outcomes of patients with acute ischemic stroke measured by BI [39]. On hospital admission, a BI less than 65 predicts mortality within six months of discharge. Functional disability on admission was predictive of institutionalization on discharge [40]. In a Danish nationwide population-based cohort study, including patients aged ≥65 years admitted to the geriatric departments, BI at admission is strongly and independently associated with mortality [41]. In this study, BI did not enter the multivariate analysis as an independent predictor of mortality, even if the Barthel score clearly identified in-patients with diabetes in comparison with other patients without diabetes. According to our analysis, an increase of 10 points in the BI score was protective by 21% from the risk of death. It is, therefore, logical to assert that BI is one of the strongest predictors of mortality in diabetic patients too. Concerning the role of a higher glomerular filtration rate, diabetic nephropathy occurs in up to 50% of people with diabetes, and represents a major cause of end-stage kidney disease, and is associated with significantly increased cardiovascular morbidity and mortality [42]. A recent population-based cohort study from primary care UK electronic health records showed higher all-cause mortality risks in subjects with older age, and reduced renal function among people with type 2 diabetes [43]. In our analysis, an increase of 10 mL/min of glomerular filtration rate was protective by 15% from the risk of death. We found that peripheral artery disease had a protective role regarding 1-year mortality. It is well known that diabetes mellitus increases the incidence of peripheral artery disease, accelerates disease progression, and increases disease severity. Patients with concomitant diabetes mellitus and peripheral artery disease are at high risk for major complications, such as amputation. The EUCLID trial showed that patients with peripheral artery disease are at higher risk of cardiovascular events: every 1% increase in HbA1c was associated with a 14.2% increased relative risk for MACE [44]. According to a recent meta-analysis, diabetes is associated with an increased risk of mortality in peripheral vascular disease, particularly in patients with critical limb ischemia [45]. A possible explanation lies in the early therapeutic measures provided by clinicians that did not make these patients more susceptible to ischemic events and functional impairment, significantly improving daily quality of life, cardiovascular risk, and patient outcomes. Most of these patients were treated with physical exercise, and it is well known that physical exercise has a protector effect. This effect may be justified by a greater development of collateral circulation, which is currently one of the therapeutic recommendations in these kinds of patients [46,47]. This study had some limitations. First, no specific information about diabetes duration is available. Second, HbA1c, which is the better indicator of chronic glycemic levels and risk for long-term complications, is lacking. Third, the RePoSi registry was not specifically designed to evaluate clinical information. The major strength of the study is the multicenter design of the RePoSi, with a population consisting of hospitalized elderly patients in internal medicine and geriatric wards with multiple and more severe diseases.

## 5. Conclusions

In conclusion, this study showed the impact of diabetes and its comorbidities in the elderly patients admitted in internal and geriatric wards. Moreover, our findings highlight factors related to in-hospital and follow-up outcomes in this population. Our results provide data for a better tailored management, and for the creation of health care pathways from the hospital to tertiary care, allowing us to identify subjects with diabetes at higher risk at the admission to the hospital, and at the post-discharge follow-up. This approach should be mandatory for a correct management based on a comprehensive assessment of elderly patients affected by diabetes.

## Figures and Tables

**Table 1:** Socio-demographic characteristics and some modifiable risk factors of the RePoSi elderly population according to diabetes categorization.

Variables	Inpatient with Diabetes	Inpatient without Diabetes	*p*
N° of subjects	1378	3330	/
Men (%)	56.2	43.8	<0.0001
Age *	78.45 (78.05–78.85)	79.68 (79.40–79.96)	<0.0001
Marital status (%)			0.283
Married	56.5	52.5	
Widow	33.5	37	
Separated	1.3	1.1	
Divorced	1.3	1.4	
Living arrangement (%)			0.042
Alone	20.1	24.2	
Spouse	46.6	44.4	
Sons	15.5	15.2	
Spouse and sons	8.8	6.7	
Other	8.4	9.4	
Previously Institutionalized (%)	6.7	5.3	0.0853
Previously Hospitalized (%)	43.0	34.5	0.0046
Caregiver (%)	54.5	52.9	0.3673
Spouse (%)	38.0	32.2	0.168
Brother/Sister (%)	3.5	3.5	
Son/Daughter (%)	44.6	47.9	
Son/Daughter in law (%)	1.3	1.2	
Grandson (%)	3.7	4.0	
Other (%)	8.9	11.2	
Never smoked (%)	49.3	56.1	<0.0001
ex-Smoker (%)	42.4	35.1	
Smoker (%)	8.3	8.9	
Alcohol (%)	64.4	60.8	0.0346
BMI *	27.40 (27.06–27.75)	25.27 (25.09–25.45)	<0.0001
Waist circumference men (cm) *	98.03 (95.84–100.22)	94.53 (93.15–95.91)	0.0242
Waist circumference women (cm) *	98.37 (95.55–101.19)	89.75 (88.35–91.15)	<0.0001
Visceral obesity men (%)	33.5	27.8	0.1544
Visceral obesity women (%)	79.7	56.8	<0.0001
Underweight patients (%)	2.0	4.4	0.0004
Optimal weight patients (%)	31.5	45	<0.0001
Overweight patients (%)	39.0	34.4	0.0080
Class I obesity (%)	17.2	10.9	<0.0001
Class II obesity (%)	5.3	2.2	<0.0001
Class III obesity (%)	2.8	0.8	<0.0001

* Data are reported as mean (95% confidence interval).

**Table 2:** Laboratory and clinical characteristics of the RePoSi population at hospital admission according to diabetes categorization.

Variables	Inpatient with Diabetes	Inpatient without Diabetes	*p*
Systolic blood pressure (mm Hg) *	132.23 (131.0–133.4)	131.5 (130.7–132.3)	0.2247
Diastolic blood pressure (mm Hg) *	73.5 (72.8–74.1)	73.7 (73.3–74.2)	0.7048
Heart rate (bpm) *	78.5 (77.6–79.4)	79.1 (78.5–79.8)	0.6417
Body temperature (°C) *	36.9 (36.8–36.9)	36.9 (36.9–37.0)	0.0566
Fasting glucose (mg/dL) *	167.1 (162–172.3)	110.4 (109.0–111.9)	<0.0001
Creatinine (mg/dL) *	1.4 (1.3–1.4)	1.2 (1.2–1.2)	<0.0001
Glomerular Filtration Rate *	55.9 (54.5–57.3)	61.2 (60.4–62.1)	<0.0001
Hemoglobin (mg/dL) *	11.7 (11.6–11.9)	11.9 (11.8–12.0)	0.0022
Leucocytes (cells per microliter) * (×103/uL)	9.7 (9.1–10.2)	10 (9.1–10.8)	0.0258
Platelets (cells per microliter) * (×103/uL)	230.0 (222.5–237.4)	231.2 (227.2–235.1)	0.4135
Cholesterol (mg/dL) *	154.2 (151.1–157.3)	163.2 (161.2–165.1)	<0.0001
Short Blessed Test score *	9.4 (8.9–9.8)	9.2 (8.9–9.5)	0.4136
Overt Cognitive impairment (Short Blessed Test score ≥ 10) (%)	36.2	36.5	0.8481
Need for urinary catheter (%)	29.3	24.6	0.0023
Barthel index score *	76.7 (75,0–78.4)	78.3 (77.1–79.4)	0.0019
Clinically significant disability (Barthel index ≤ 40) (%)	15.4	14.3	0.3627
Geriatric Depression Scale score *	1.4 (1.3–1.5)	1.4 (1.3–1.4)	0.9104
Probable Depression (Geriatric Depression Scale score > 2) (%)	18.1	18.4	0.8553
N° of drugs at hospital admission *	7.1 (6.9–7.3)	5.1 (5.0–5.2)	<0.0001
N° of in-hospital drugs *	8.9 (8.5–9.2)	6.9 (6.7–7.1)	<0.0001
N° of drugs at hospital discharge *	8.8 (8.6–9.1)	6.8 (6.6–6.9)	<0.0001
N° of drugs at follow up 3 months *	7.8 (7.5–8.0)	5.9 (5.8–6.1)	<0.0001
N° of drugs at follow up 1 year *	8.0 (7.5–8.5)	5.7 (5.4–6.1)	<0.0001
Severity index (by CIRS) *	1.80 (1.78–1.81)	1.60 (1.59–1.61)	<0.0001
Comorbidity index (by CIRS) *	3.81 (3.69–3.92)	2.69 (2.62–2.75)	<0.0001

* Data are reported as mean (95% confidence interval); BMI = body mass index; CIRS = cumulative illness rating scale.

**Table 3:** Length of hospital stay, destination at hospital discharge, and in-hospital and at follow-up mortality of the whole RePoSi population according to diabetes categorization.

Variables	Inpatient with Diabetes	Inpatient without Diabetes	*p*
Length of hospital stay * (days)	11.9 (11.2–12.5)	11.7 (11.2–12.1)	0.5836
In-hospital mortality (%)	4.6	3.9	0.2853
1-year mortality (%)	42.9	39.0	0.2644

* Data are reported as means (95% confidence interval).

## Appendix A. Investigators and Co-Authors of the RePoSi (REgistro POliterapie SIMI, Società Italiana di Medicina Interna) Study Group are as Follows

Steering Committee: Pier Mannuccio Mannucci (Chair) (Fondazione IRCCS Cà Granda Ospedale Maggiore Policlinico, Milano), Alessandro Nobili (Co-chair) (Istituto di Ricerche Farmacologiche Mario Negri IRCCS, Milano), Antonello Pietrangelo (Presidente SIMI), Francesco Perticone (Direttore CRIS–SIMI), Giuseppe Licata (Socio d’onore SIMI), Francesco Violi (Policlinico Umberto I, Roma, Prima Clinica Medica), Gino Roberto Corazza, (Reparto 11, IRCCS Policlinico San Matteo di Pavia, Pavia, Clinica Medica I), Salvatore Corrao (ARNAS Civico, Di Cristina, Benfratelli, DiBiMIS, Università di Palermo, Palermo), Alessandra Marengoni (Spedali Civili di Brescia, Brescia), Francesco Salerno (IRCCS Policlinico San Donato Milanese, Milano), Matteo Cesari (UO Geriatria, Università degli Studi di Milano), Mauro Tettamanti, Luca Pasina, Carlotta Franchi (Istituto di Ricerche Farmacologiche Mario Negri IRCCS, Milano).

Clinical data monitoring and revision: Carlotta Franchi, Laura Cortesi, Mauro Tettamanti, Gabriella Miglio (Istituto di Ricerche Farmacologiche Mario Negri IRCCS, Milano).

Database Management and Statistics: Mauro Tettamanti, Laura Cortesi, Ilaria Ardoino, Alessio Novella (Istituto di Ricerche Farmacologiche Mario Negri IRCCS, Milano).

Investigators:

Domenico Prisco, Elena Silvestri, Giacomo Emmi, Alessandra Bettiol, Irene Mattioli (Azienda Ospedaliero Universitaria Careggi Firenze, Medicina Interna Interdisciplinare).

1. Gianni Biolo, Michela Zanetti, Giacomo Bartelloni (*Azienda Sanitaria Universitaria Integrata di Trieste*, *Clinica Medica Generale e Terapia Medica*);
2. Massimo Vanoli, Giulia Grignani, Edoardo Alessandro Pulixi (*Azienda Ospedaliera della Provincia di Lecco*, *Ospedale di Merate*, *Lecco*, *Medicina Interna*);
3. Graziana Lupattelli, Vanessa Bianconi, Riccardo Alcidi (*Azienda Ospedaliera Santa Maria della Misericordia*, *Perugia*, *Medicina Interna*);
4. Domenico Girelli, Fabiana Busti, Giacomo Marchi (*Azienda Ospedaliera Universitaria Integrata di Verona*, *Verona*, *Medicina Generale e Malattie Aterotrombotiche e Degenerative*);
5. Mario Barbagallo, Ligia Dominguez, Vincenza Beneduce, Federica Cacioppo (*Azienda Ospedaliera Universitaria Policlinico Giaccone Policlinico di Palermo*, *Palermo*, *Unità Operativa di Geriatria e Lungodegenza*);
6. Salvatore Corrao, Giuseppe Natoli, Salvatore Mularo, Massimo Raspanti, Christiano Argano (*A*.*R*.*N*.*A*.*S*. *Civico*, *Di Cristina*, *Benfratelli*, *Palermo*, *UOC Medicina Interna ad Indirizzo Geriatrico-Riabilitativo*);
7. Marco Zoli, Maria Laura Matacena, Giuseppe Orio, Eleonora Magnolfi, Giovanni Serafini, Angelo Simili (*Azienda Ospedaliera Universitaria Policlinico S*. *Orsola-Malpighi*, *Bologna*, *Unità Operativa di Medicina Interna*);
8. Giuseppe Palasciano, Maria Ester Modeo, Carla Di Gennaro (*Azienda Ospedaliero**-Universitaria Consorziale Policlinico di Bari*, *Bari*, *Medicina Interna Ospedaliera “L*. *D’Agostino”*, *Medicina Interna Universitaria “A*. *Murri”*);
9. Maria Domenica Cappellini, Giovanna Fabio, Margherita Migone De Amicis, Giacomo De Luca, Natalia Scaramellini (*Fondazione IRCCS Cà Granda Ospedale Maggiore Policlinico*, *Milano*, *Unità Operativa Medicina Interna IA*);
10. Matteo Cesari, Paolo Dionigi Rossi, Sarah Damanti, Marta Clerici, Simona Leoni, Alessandra Danuta Di Mauro (*Fondazione IRCCS Cà Granda Ospedale Maggiore Policlinico*, *Milano*, *Geriatria*);
11. Antonio Di Sabatino, Emanuela Miceli, Marco Vincenzo Lenti, Martina Pisati, Costanza Caccia Dominioni (*IRCCS Policlinico San Matteo di Pavia*, *Pavia*, *Clinica Medica I*, *Reparto 11*);
12. Roberto Pontremoli, Valentina Beccati, Giulia Nobili, Giovanna Leoncini (*IRCCS Azienda Ospedaliera Universitaria San Martino-IST di Genova*, *Genova*, *Clinica di Medicina Interna 2*);
13. Luigi Anastasio, Maria Carbone (*Ospedale Civile Jazzolino di Vibo Valentia*, *Vibo Valentia*, *Medicina interna*);
14. Francesco Cipollone, Maria Teresa Guagnano, Ilaria Rossi (*Ospedale Clinicizzato SS*. *Annunziata*, *Chieti*, *Clinica Medica*);
15. Gerardo Mancuso, Daniela Calipari, Mosè Bartone (*Ospedale Giovanni Paolo II Lamezia Terme*, *Catanzaro*, *Unità Operativa Complessa Medicina Interna*);
16. Giuseppe Delitala, Maria Berria, Alessandro Delitala (*Azienda ospedaliera-universitaria di Sassari*, *Clinica Medica*);
17. Maurizio Muscaritoli, Alessio Molfino, Enrico Petrillo, Antonella Giorgi, Christian Gracin (*Policlinico Umberto I*, *Sapienza Università di Roma*, *Medicina Interna e Nutrizione Clinica Policlinico Umberto I*);
18. Giuseppe Zuccalà, Gabriella D’Aurizio (*Policlinico Universitario A*. *Gemelli*, *Roma*, *Roma*, *Unità Operativa Complessa Medicina d’Urgenza e Pronto Soccorso*);
19. Giuseppe Romanelli, Alessandra Marengoni, Andrea Volpini, Daniela Lucente (*Unità Operativa Complessa di Medicina I a indirizzo geriatrico*, *Spedali Civili*, *Montichiari* (*Brescia*));
20. Antonio Picardi, Umberto Vespasiani Gentilucci, Paolo Gallo (*Università Campus Bio-Medico*, *Roma*, *Medicina Clinica-Epatologia*);
21. Giuseppe Bellelli, Maurizio Corsi, Cesare Antonucci, Chiara Sidoli, Giulia Principato (*Università degli studi di Milano-Bicocca Ospedale S*. *Gerardo*, *Monza*, *Unità Operativa di Geriatria*);
22. Franco Arturi, Elena Succurro, Bruno Tassone, Federica Giofrè (*Università degli Studi Magna Grecia*, *Policlinico Mater Domini*, *Catanzaro*, *Unità Operativa Complessa di Medicina Interna*);
23. Maria Grazia Serra, Maria Antonietta Bleve (*Azienda Ospedaliera “Cardinale Panico” Tricase*, *Lecce*, *Unità Operativa Complessa Medicina*);
24. Antonio Brucato, Teresa De Falco (*ASST Fatebenefratelli–Sacco*, *Milano*, *Medicina Interna*);
25. Fabrizio Fabris, Irene Bertozzi, Giulia Bogoni, Maria Victoria Rabuini, Tancredi Prandini (*Azienda Ospedaliera Università di Padova*, *Padova*, *Clinica Medica I*);
26. Roberto Manfredini, Fabio Fabbian, Benedetta Boari, Alfredo De Giorgi, Ruana Tiseo (*Azienda Ospedaliera–Universitaria Sant’Anna*, *Ferrara*, *Unità Operativa Clinica Medica*);
27. Giuseppe Paolisso, Maria Rosaria Rizzo, Claudia Catalano (*Azienda Ospedaliera* *Universitaria della Seconda Università degli Studi di Napoli*, *Napoli*, *VI Divisione di Medicina Interna e Malattie Nutrizionali dell’Invecchiamento*);
28. Claudio Borghi, Enrico Strocchi, Eugenia Ianniello, Mario Soldati, Silvia Schiavone, Alessio Bragagni (*Azienda Ospedaliera Universitaria Policlinico S*. *Orsola-Malpighi*, *Bologna*, *Unità Operativa di Medicina Interna Borghi*);
29. Carlo Sabbà, Francesco Saverio Vella, Patrizia Suppressa, Giovanni Michele De Vincenzo, Alessio Comitangelo, Emanuele Amoruso, Carlo Custodero (*Azienda Ospedaliero-Universitaria Consorziale Policlinico di Bari*, *Bari*, *Medicina Interna Universitaria C*. *Frugoni*);
30. Luigi Fenoglio, Andrea Falcetta (*Azienda Sanitaria Ospedaliera Santa Croce e Carle di Cuneo*, *Cuneo*, *S*. *C*. *Medicina Interna*);
31. Anna L. Fracanzani, Silvia Tiraboschi, Annalisa Cespiati, Giovanna Oberti, Giordano Sigon (*Fondazione IRCCS Cà Granda Ospedale Maggiore Policlinico*, *Milano*, *Medicina Interna 1B*);
32. Flora Peyvandi, Raffaella Rossio, Giulia Colombo, Pasquale Agosti (*Fondazione IRCCS Cà Granda Ospedale Maggiore Policlinico*, *Milano*, *UOC Medicina generale–Emostasi e trombosi*);
33. Valter Monzani, Valeria Savojardo, Giuliana Ceriani (*Fondazione IRCCS Cà Granda Ospedale Maggiore Policlinico*, *Milano*, *Medicina Interna Alta Intensità*);
34. Francesco Salerno, Giada Pallini (*IRCCS Policlinico San Donato e Università di Milano*, *San Donato Milanese*, *Medicina Interna*);
35. Fabrizio Montecucco, Luciano Ottonello, Lara Caserza, Giulia Vischi (*IRCCS Ospedale Policlinico San Martino e Università di Genova*, *Genova*, *Medicina Interna 1*);
36. Nicola Lucio Liberato, Tiziana Tognin (*ASST di Pavia*, *UOSD Medicina Interna*, *Ospedale di Casorate Primo*, *Pavia*);
37. Francesco Purrello, Antonino Di Pino, Salvatore Piro (*Ospedale Garibaldi Nesima*, *Catania*, *Unità Operativa Complessa di Medicina Interna*);
38. Renzo Rozzini, Lina Falanga, Maria Stella Pisciotta, Francesco Baffa Bellucci, Stefano Buffelli (*Ospedale Poliambulanza*, *Brescia*, *Medicina Interna e Geriatria*);
39. Giuseppe Montrucchio, Paolo Peasso, Edoardo Favale, Cesare Poletto, Carl Margaria, Maura Sanino (Dipartimento di Scienze Mediche, Università di Torino, Città della Scienza e della Salute, Torino, Medicina Interna 2 U. Indirizzo d’Urgenza);
40. Francesco Violi, Ludovica Perri (*Policlinico Umberto I*, *Roma*, *Prima Clinica Medica*);
41. Luigina Guasti, Luana Castiglioni, Andrea Maresca, Alessandro Squizzato, Leonardo Campiotti, Alessandra Grossi, Roberto Davide Diprizio (*Università degli Studi dell’Insubria*, *Ospedale di Circolo e Fondazione Macchi*, *Varese*, *Medicina Interna I*);
42. Marco Bertolotti, Chiara Mussi, Giulia Lancellotti, Maria Vittoria Libbra, Matteo Galassi, Yasmine Grassi, Alessio Greco (*Università di Modena e Reggio Emilia*, *Azienda Ospedaliero-Universitaria di Modena*; *Ospedale Civile di Baggiovara*, *Unità Operativa di Geriatria*);
43. Angela Sciacqua, Maria Perticone, Rosa Battaglia, Raffaele Maio (*Università Magna Grecia Policlinico Mater Domini*, *Catanzaro*, *Unità Operativa Malattie Cardiovascolari Geriatriche*);
44. Vincenzo Stanghellini, Eugenio Ruggeri, Sara del Vecchio (*Dipartimento di Scienze Mediche e Chirurgiche*, *Unità Operativa di Medicina Interna*, *Università degli Studi di Bologna/Azienda Ospedaliero-Universitaria S*.*Orsola-Malpighi*, *Bologna*);
45. Andrea Salvi, Roberto Leonardi, Giampaolo Damiani (*Spedali Civili di Brescia*, *U*.*O*. *3a Medicina Generale*);
46. William Capeci, Massimo Mattioli, Giuseppe Pio Martino, Lorenzo Biondi, Pietro Pettinari (*Clinica Medica*, *Azienda Ospedaliera Universitaria–Ospedali Riuniti di Ancona*);
47. Riccardo Ghio, Anna Dal Col (*Azienda Ospedaliera Università San Martino*, *Genova*, *Medicina III*);
48. Salvatore Minisola, Luciano Colangelo, Mirella Cilli, Giancarlo Labbadia (*Policlinico Umberto I*, *Roma*, *SMSC03–Medicina Interna A e Malattie Metaboliche dell’osso*);
49. Antonella Afeltra, Benedetta Marigliano, Maria Elena Pipita (*Policlinico Campus Biomedico Roma*, *Roma*, *Medicina Clinica*);
50. Pietro Castellino, Luca Zanoli, Alfio Gennaro, Agostino Gaudio (*Azienda Ospedaliera Universitaria Policlinico–V*. *Emanuele*, *Catania*, *Dipartimento di Medicina*);
51. Valter Saracco, Marisa Fogliati, Carlo Bussolino (*Ospedale Cardinal Massaia Asti*, *Medicina A*);
52. Francesca Mete, Miriam Gino (*Ospedale degli Infermi di Rivoli*, *Torino*, *Medicina Interna*);
53. Carlo Vigorito, Antonio Cittadini, (*Azienda Policlinico Universitario Federico II di Napoli*, *Napoli*, *Medicina Interna e Riabilitazione Cardiologica*);
54. Guido Moreo, Silvia Prolo, Gloria Pina (*Clinica San Carlo Casa di Cura Polispecialistica*, *Paderno Dugnano*, *Milano*, *Unità Operativa di Medicina Interna*);
55. Alberto Ballestrero, Fabio Ferrando, Roberta Gonella, Domenico Cerminara (*Clinica Di Medicina Interna ad Indirizzo Oncologico*, *Azienda Ospedaliera Università San Martino di Genova*);
56. Sergio Berra, Simonetta Dassi, Maria Cristina Nava (*Medicina Interna*, *Azienda Ospedaliera Guido Salvini*, *Garnagnate*, *Milano*);
57. Bruno Graziella, Stefano Baldassarre, Salvatore Fragapani, Gabriella Gruden (*Medicina Interna III*, *Ospedale S*. *Giovanni Battista Molinette*, *Torino*);
58. Giorgio Galanti, Gabriele Mascherini, Cristian Petri, Laura Stefani (*Agenzia di Medicina dello Sport*, *AOUC Careggi*, *Firenze*);
59. Margherita Girino, Valeria Piccinelli (Medicina Interna, Ospedale S. Spirito Casale Monferrato, Alessandria);
60. Francesco Nasso, Vincenza Gioffrè, Maria Pasquale (*Struttura Operativa Complessa di Medicina Interna*, *Ospedale Santa Maria degli Ungheresi*, *Reggio Calabria*);
61. Leonardo Sechi, Cristiana Catena, Gianluca Colussi, Alessandro Cavarape, Andea Da Porto (*Clinica Medica*, *Azienda Ospedaliera Universitaria*, *Udine*);
62. Nicola Passariello, Luca Rinaldi (*Presidio Medico di Marcianise*, *Napoli*, *Medicina Interna*);
63. Franco Berti, Giuseppe Famularo, Patrizia Tarsitani (*Azienda Ospedaliera San Camillo Forlanini*, *Roma*, *Medicina Interna II*);
64. Roberto Castello, Michela Pasino (*Ospedale Civile Maggiore Borgo Trento*, *Verona*, *Medicina Generale e Sezione di Decisione Clinica*);
65. Gian Paolo Ceda, Marcello Giuseppe Maggio, Simonetta Morganti, Andrea Artoni, Margherita Grossi (*Azienda Ospedaliero Universitaria di Parma*, *U*.*O*.*C Clinica Geriatrica*);
66. Stefano Del Giacco, Davide Firinu, Giulia Costanzo, Giacomo Argiolas (*Policlinico Universitario Duilio Casula*, *Azienda Ospedaliero-Universitaria di Cagliari*, *Cagliari*, *Medicina Interna*, *Allergologia ed Immunologia Clinica*);
67. Giuseppe Montalto, Anna Licata, Filippo Alessandro Montalto (*Azienda Ospedaliera Universitaria Policlinico Paolo Giaccone*, *Palermo*, *UOC di Medicina Interna*);
68. Francesco Corica, Giorgio Basile, Antonino Catalano, Federica Bellone, Concetto Principato (*Azienda Ospedaliera Universitaria Policlinico G*. *Martino*, *Messina*, *Unità Operativa di Geriatria*);
69. Lorenzo Malatino, Benedetta Stancanelli, Valentina Terranova, Salvatore Di Marca, Rosario Di Quattro, Lara La Malfa, Rossella Caruso (Azienda Ospedaliera per l’Emergenza Cannizzaro, Catania, Clinica Medica Università di Catania);
70. Patrizia Mecocci, Carmelinda Ruggiero, Virginia Boccardi (*Università degli Studi di Perugia-Azienda Ospedaliera S*.*M*. *della Misericordia*, *Perugia*, *Struttura Complessa di Geriatria*);
71. Tiziana Meschi, Andrea Ticinesi, Antonio Nouvenne (*Azienda Ospedaliera Universitaria di Parma*, *U*.*O Medicina Interna e Lungodegenza Critica*);
72. Pietro Minuz, Luigi Fondrieschi, Giandomenico Nigro Imperiale (*Azienda Ospedaliera Universitaria Verona*, *Policlinico GB Rossi*, *Verona*, *Medicina Generale per lo Studio ed il Trattamento dell’Ipertensione Arteriosa*);
73. Mario Pirisi, Gian Paolo Fra, Daniele Sola, Mattia Bellan (*Azienda Ospedaliera Universitaria Maggiore della Carità*, *Medicina Interna 1*);
74. Massimo Porta, Piero Riva (*Azienda Ospedaliera Universitaria Città della Salute e della Scienza di Torino*, *Medicina Interna 1U*);
75. Roberto Quadri, Erica Larovere, Marco Novelli (*Ospedale di Ciriè*, *ASL TO4*, *Torino*, *S*.*C*. *Medicina Interna*);
76. Giorgio Scanzi, Caterina Mengoli, Stella Provini, Laura Ricevuti (*ASST Lodi*, *Presidio di Codogno*, *Milano*, *Medicina*);
77. Emilio Simeone, Rosa Scurti, Fabio Tolloso (*Ospedale Spirito Santo di Pescara*, *Geriatria*);
78. Roberto Tarquini, Alice Valoriani, Silvia Dolenti, Giulia Vannini (*Ospedale San Giuseppe*, *Empoli*, *USL Toscana Centro*, *Firenze*, *Medicina Interna I*);
79. Riccardo Volpi, Pietro Bocchi, Alessandro Vignali (*Azienda Ospedaliera Universitaria di Parma*, *Clinica e Terapia Medica*);
80. Sergio Harari, Chiara Lonati, Federico Napoli, Italia Aiello (*Ospedale San Giuseppe Multimedica Spa*, *U*.*O*. *Medicina Generale*);
81. Raffaele Landolfi, Massimo Montalto, Antonio Mirijello (*Policlinico Universitario A*. *Gemelli- Roma*, *Clinica Medica*);
82. Francesco Purrello, Antonino Di Pino (*Ospedale Garibaldi–Nesima–Catania*, *U*.*O*.*C Medicina Interna*);
83. Nome e Cognome del Primario, Silvia Ghidoni (*Azienda Ospedaliera Papa Giovanni XXIII*, *Bergamo*, *Medicina I*);
84. Teresa Salvatore, Lucio Monaco, Carmen Ricozzi (*Policlinico Università della Campania L*. *Vanvitelli*, *UOC Medicina Interna*);
85. Alberto Pilotto, Ilaria Indiano, Federica Gandolfo (*Ente Ospedaliero Ospedali Galliera Genova*, *SC Geriatria Dipartimento Cure Geriatriche*, *Ortogeriatria e Riabilitazione*).
